# Insights into Factors Affecting Traffic Accident Severity of Novice and Experienced Drivers: A Machine Learning Approach

**DOI:** 10.3390/ijerph182312725

**Published:** 2021-12-02

**Authors:** Shuaiming Chen, Haipeng Shao, Ximing Ji

**Affiliations:** College of Transportation Engineering, Chang’an University, Xi’an 710064, China; chenshuaiming@chd.edu.cn (S.C.); jiximing@chd.edu.cn (X.J.)

**Keywords:** traffic safety, accident severity, driving experience, machine learning, CatBoost

## Abstract

Traffic accidents have significant financial and social impacts. Reducing the losses caused by traffic accidents has always been one of the most important issues. This paper presents an effort to investigate the factors affecting the accident severity of drivers with different driving experience. Special focus was placed on the combined effect of driving experience and age. Based on our dataset (traffic accidents that occurred between 2005 and 2021 in Shaanxi, China), CatBoost model was applied to deal with categorical feature, and SHAP (Shapley Additive exPlanations) model was used to interpret the output. Results show that accident cause, age, visibility, light condition, season, road alignment, and terrain are the key factors affecting accident severity for both novice and experienced drivers. Age has the opposite impact on fatal accident for novice and experienced drivers. Novice drivers younger than 30 or older than 55 are prone to suffer fatal accident, but for experienced drivers, the risk of fatal accident decreases when they are young and increases when they are old. These findings fill the research gap of the combined effect of driving experience and age on accident severity. Meanwhile, it can provide useful insights for practitioners to improve traffic safety for novice and experienced drivers.

## 1. Introduction

According to China Statistical Yearbook (2020), in 2019, there were 247,646 road traffic accidents in China, resulting in 62,763 deaths, 25,101 injuries, and direct property losses of 1346.179 million CNY. The harm of traffic accidents to human and society is self-evident; in order to reduce the occurrence of traffic accidents, it is necessary to explore the factors affecting the severity of accidents. Driving experience is the key factor that cannot be ignored in accident analysis. Studies show that novice drivers are prone to suffer fatal accidents [1,2]. In addition, compared with experienced drivers, novice drivers tend to overestimate driving skills [3] and more likely to be disturbed by external factors, such as smartphones and billboards, which makes novice drivers more vulnerable to serious injury. Therefore, it is necessary to investigate and analyze the influencing factors of accident severity for novice drivers and experienced drivers.

At present, many scholars have conducted research on accident analysis of drivers with different driving experience. Xiao et al. [4] found that there is correlation between novice and experienced drivers for influencing factors of accident severity, but the correlation is not strong. Al Garawi’s study [5] of novice female drivers with different ages found no significant difference in accident rates among very young females, intermediate females, and older females. Young drivers are more prone to suffer accidents than middle-aged drivers in different groups with significant difference in driving experience. Moral García [6] found that in accidents involving novice drivers, speeding is a key factor; besides, poor road condition and roads without sidewalks also make novice drivers more prone to serious accidents. However, these studies mostly focus on a certain driving experience group or the independent influence of a single factor, ignoring the comparison of different driving experience groups and the combined effect of factors. In this study, drivers are divided into three groups according to their driving experience, and the influencing factors of accident severity of different groups are comparatively studied. On this basis, the combined effect of age and driving experience is analyzed. The CatBoost (Categorical Boosting) model is applied to deal with many categorical features in accident data. As a boosting algorithm, CatBoost can deal with categorical features well and has superior performance compared with XGBoost (eXtreme Gradient Boosting) and LightGBM (Light Gradient Boosting Machine) [7], which has been widely used in computer vision, data mining, and other fields. However, the difficulty in applying machine learning methods is how to clearly interpret the results. In this paper, SHAP (Shapley Additive exPlanations) model is used to explain the CatBoost model output. SHAP is based on game theory and interprets the model by calculating each variable’s contribution to the prediction, and one study shows that it can be used to interpret any machine learning model [8].

The paper is organized as follows: Section 2 reviews the research on the traffic accident severity of drivers with different driving experience and related models. Section 3 lists the data sources in detail and analyzes the accident distribution characteristics. Section 4 introduces the methods used in this paper, and Section 5 analyzes the model results and discusses the key results obtained from the model. Section 6 draws the conclusions. The innovation of our study is analyzing the combined influence of driving experience and age on traffic accident severity. The key questions to be addressed are: what are the major factors causing serious accident outcomes for drivers with different driving experience? Are there any significant variations in their impacts?

## 2. Literature Review

### 2.1. Novice and Experienced Driver in Traffic Accident Analysis

Previous studies [9,10] showed that driving experience is an important factor in accident analysis, and the novice driver is prone to being fatally injured. Traffic accident analysis for novice drivers and experienced drivers is one of the hot spots in traffic safety research.

Through simulation experiment and questionnaire survey, Ivers et al. [11] found that dangerous driving behavior of novice drivers is related to the increase of accident risk. Craen et al. [3] also found that novice drivers tend to overestimate their driving skills. Through driving simulation experiment, Ohlhauser et al. [12] found that the PRT (Perception Response Time) of novice drivers was significantly longer than that of experienced drivers. Moral García et al. [6] studied the traffic accidents of novice driver in urban areas using the decision tree ensemble method, and the results showed that speeding is the main cause of serious injury.

Compared with novice drivers, experienced drivers perform better in driving experience, decision-making ability, and other aspects. The research on experienced drivers focuses on the comparison with novice drivers at present. Mitchell et al. [13] compared common collision accidents between novice drivers and experienced drivers, and they found that the accident characteristics of novice drivers and experienced drivers were similar, but speeding, drowsy driving, and drunk driving were significant factors causing accidents for novice drivers. By analyzing the eye movement data of novice and experienced drivers when driving on different types of roads, Underwood et al. [14] found that compared with novice drivers, experienced drivers showed higher sensitivity on the whole. Xiao et al. [4] used the bivariate random-effects probit model to analyze the influencing factors of accident severity for novice and experienced drivers, and they found that the key factors of fatal injury suffered by novice drivers and experienced drivers are different.

### 2.2. Traffic Accident Severity Modeling

Discrete response models are widely used in the modeling of accident severity in previous studies. Bedard et al. [15] applied multivariate logistic regression to evaluate the influence of the driver, accident, and vehicle characteristics on fatal injury, and found that older drivers, females, not wearing seat belt, and speeding would lead to serious accidents. However, the disordered response model fails to reflect the internal order of the accident severity variable, and scholars use the ordered response model instead [16,17]. In addition, the generalized ordered model [18,19,20] and random parameter model [21,22] are also introduced. The generalized ordered model is an improvement of the ordered model; it believes that the external variables have different effects on different alternatives, while the random parameter model believes that the parameters in the model are random. Shao et.al [23] analyzed the factors affecting the severity of truck involved rear-end collisions. They found that there is a significant difference between the car-strike-truck and truck-strike-car crashes. Chen et.al [24] found a significant correlation between the severity of injuries of two drivers in the same rear-end collision. Driver’s age, gender, vehicle type, and use of airbag or safety belt are found to affect injury severity. In addition, road attributes, such as road surface condition and road width, are also closely related to accident severity. Satoshi’s study [25] showed that snow-covered road surface conditions significantly reduced the severity of accident. Xiao et al. [26] found that narrow lanes (8 ft~11 ft) increase the collision risk; for sections with many lanes, a lane width of no less than 12 ft helps to reduce the risk. When studying the factor affecting the severity of truck and passenger car, Zhou et al. [27] found that non-intersection areas are more prone to suffer fatal accidents. The research results of Ma [16] showed that when hazardous material transportation accidents occur on highway, the probability of fatal accidents is higher than that of other road types.

In the past two decades, the rapid development and excellent performance of machine learning methods have attracted extensive attention of researchers. Compared with traditional statistical methods, machine learning methods have higher flexibility, almost no presupposition on accident severity data, and can deal with missing values and noise [28]. Li et al. [29] used SVM to analyze the injury severity and found that SVM model was superior to ordered probit model in accuracy. Yu et al. [30] used CART model to select variables before modeling with SVM. Chen et al. [31] also used SVM model to study the injury severity in rollover accident and used CART model to identify significant variables, finding that SVM model with polynomial kernel function did better in prediction. Alkheder et al. [32] applied Decision Tree, Bayesian Network, and linear SVM to analyze the risk factors related to traffic accident severity. They found that road type and accident type are key factors.

To improve model performance, multiple weak learners can be combined to form a strong learner, that is, ensemble learning. In accident severity analysis, mainly two ensemble learning methods are used—bagging (i.e., random forest [28,33]) and boosting. Gradient boosting is an implementation of boosting method that could achieve most advanced results in a variety of practical tasks. It has been the primary method during the past decades for solving learning problems with heterogeneous characteristics, noisy data, and complex dependency. Zhou et al. [27] used five classification models, including multinomial logistic regression, naïve bayes, CART, SVM, and XGBoost to analyze the factors affecting the severity of passenger car and truck accidents. The results showed that XGBoost combined with cost sensitive learning had the best effect. Xiao et al. [26] analyzed vehicle accidents in Texas by using LightGBM model and found that speed limit, numbers of lanes, road level, shoulder width, and shoulder type are key factors and the importance of factors varies with accident type. However, the models mentioned above will cause “dimension disaster” when dealing with the categorical features with many categories. CatBoost is a novel gradient boosting technology proposed by Yandex Company [7]. It has incomparable advantages in dealing with features with a large number of categories and is widely used in many fields but rarely used in traffic accident severity analysis.

## 3. Data Preparation

This paper collected 8447 road accidents from 2005 to 2021 in Shaanxi Province, China. A three-point ordinal scale was used to classify the severity of traffic accident, including PDO (property damage only), injury, and fatality. The distribution of the accident severity levels was as follows: PDO = 22.15%, injury = 47.78%, and fatality = 30.07%.

The definition of novice drivers in this paper was consistent with a previous study [6], and experienced drivers were divided into two groups according to their driving experience, as follows:

1. Group 1: driving experience ≤ 3 years (i.e., novice driver).
2. Group 2: 3 years < driving experience ≤ 10 years (i.e., experienced driver).
3. Group 3: driving experience > 10 years (i.e., experienced driver).

**Figure 1:**
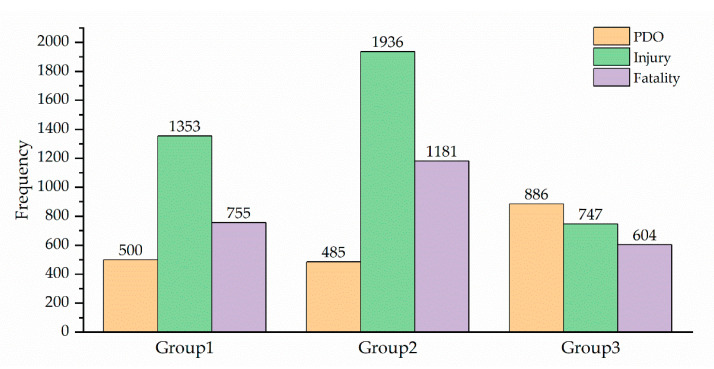
Distribution of driving experience and accident severity.

The distribution of these groups was as follows: Group 1 = 30.87%, Group 2 = 42.64%, Group 3 = 26.48%, as shown in Figure 1. A total of 16 independent variables were selected from driver properties (age, gender), vehicle properties (vehicle type, overload condition), road properties (pavement surface condition, road alignment), environment properties (day of week, season, hour, weather, visibility, traffic control, light condition, terrain), and accident properties (accident cause and accident pattern). These features are categorical features; this paper encodes the category into an ordinary number for numerical processing. The specific information is shown in Table A1.

## 4. Methodology

**Figure 2:**
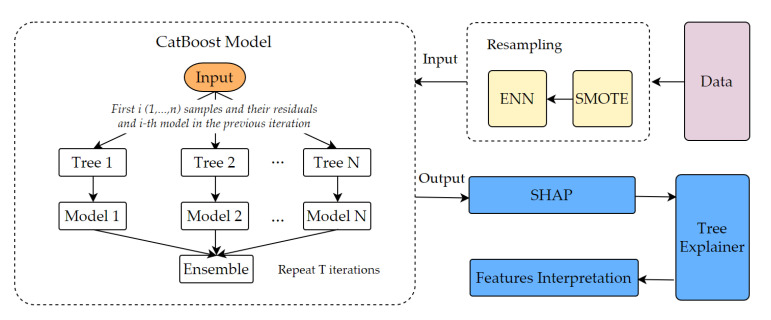
The analytic framework.

The framework of this paper is shown in Figure 2, and the methods are described in detail in this section.

### 4.1. Data Resampling

No matter what model is adopted, the inherent imbalance attribute of accident data may bring unexpected deviation. Imbalance refers to the unbalanced proportion of data in different classes. In general, the number of fatal accidents is far less than that of injury accidents. How to deal with unbalanced data is a key problem in accident severity analysis.

There are two common processing methods: over-sampling and under-sampling. Over-sampling eliminates the class imbalance by creating synthetic minority instances, including SMOTE (Synthetic Minority Over-sampling Technique) [34] and Borderline-SMOTE (BSM) [35]. Under-sampling creates better-defined class clusters by removing samples with specific selection criteria, and typical methods include ENN (Edited Nearest Neighbor) [36] and Tomeklink [37]. However, the former method increases useless information through adding samples, while the latter method losses information when removing samples. To integrate the advantages of over-sampling and over-sampling, the SMOTE-ENN method firstly uses SMOTE method to achieve over-sampling on the minority class samples and then finishes under-sampling on the majority class samples by using ENN method. This method preserves the features of majority samples and increases the characteristics of minority samples, has good classification performance for unbalanced datasets [38], and it is widely used in traffic safety analysis [39,40,41].

### 4.2. Gradient Boosting

As a classic implementation of gradient boosting, GBDT (Gradient Boosting Decision Tree) has achieved success in the field of accident severity analysis [42,43]. GBDT can be expressed as Equation (1):

(1) $$F\left(x\right)=\overset{M}{\underset{j=1}{\sum }}T\left(x;\theta \right)$$

where T(x;θ) is the decision tree; θ is the parameter of the decision tree; M is the number of trees.

The loss function of decision tree T(x;θ) is expressed as L(⋅); in GBDT, the parameter of the next decision tree is determined by minimizing the loss function, as shown in Equation (2):

(2) $$\theta_{m}=\mathrm{argmin}\overset{N}{\underset{i=1}{\sum }}L\left(y_{i},T_{m-1}\left(x\right)+T\left(x_{i};\theta \right)\right)$$

Compared with GBDT, an improvement of XGBoost is that it adds a regularization term to the objective function to reduce the complexity of the model and avoid overfitting [44]. The objective function can be expressed as Equation (3):

(3) $$L_{k}=\overset{n}{\underset{i=1}{\sum }}l\left(y^{\left(i\right)},{\hat{y}}_{k}^{\left(i\right)}\right)+\overset{k}{\underset{j=1}{\sum }}\Omega \left(f_{j}\right)$$

where n is the number of samples; l(⋅) is the loss function; ${\hat{y}}_{k}^{\left(i\right)}$ is the prediction value of the sample i at iteration k, as an additive learning approach, ${\hat{y}}_{k}^{\left(i\right)}={\hat{y}}_{k-1}^{\left(i\right)}+f_{k}\left(x^{\left(i\right)}\right)$; $f_{k}\left(\cdot \right)$ is the $k^{th}$ tree function; $\Omega \left(f_{j}\right)=\gamma T+\frac{1}{2}\lambda \overset{T}{\underset{j=1}{\sum }}\omega_{j}^{2}$ is the regularization term; T is the number of leaf nodes; γ and λ are constants.

Different from GBDT, XGBoost makes a second-order Taylor expansion of the objective function, as shown in Equation (4):

(4) $$L_{k}≅\overset{n}{\underset{i=1}{\sum }}[l\left(y_{k}^{\left(i\right)},y_{k-1}^{\left(i\right)}\right)+g^{\left(i\right)}f_{k}\left(x^{\left(i\right)}\right)+\frac{1}{2}h^{\left(i\right)}f_{k}^{2}\left(x^{\left(i\right)}\right)]+\gamma T+\frac{1}{2}\lambda \overset{T}{\underset{j=1}{\sum }}\omega_{j}^{2}$$

where $g^{\left(i\right)}$ is the first-order gradient of the loss function; $h^{\left(i\right)}$ is the second-order gradient of the loss function.

LightGBM improves the problems of GBDT and XGBoost in dealing with high-dimensional features. Different from GBDT, LightGBM uses GOSS (Gradient based One-Side Sampling) method to divide internal nodes. In GOSS, samples with large absolute value of gradient are retained, while samples with small absolute value of gradient are randomly selected to reduce the amount of calculation. In addition, LightGBM uses EFB (Exclusive Feature Bundling) method to reduce the number of features. Further explanation can be obtained in [45].

CatBoost is an implementation of Gradient Boosting Decision Trees that avoids the conditional shift with Ordered TS and the prediction shift with Ordered Boosting. Yandex proposed this algorithm in 2017 and compared it with XGBoost and LightGBM, and their empirical results show that CatBoost has a tremendous advantage over current in the boosting algorithms [7].

### 4.3. CatBoost

#### 4.3.1. Ordered TS

Generally, boosting algorithm uses one-hot encoding method to process categorical feature, but for categorical feature with many categories, this method will produce a plenty of new features. To solve this problem, categories can be grouped into limited clusters and with following application of one-hot encoding method. A common approach is to use TS (Target Statistics) to estimate the expected target value in each category and group categories based on that. That is, it uses TS feature ${\hat{x}}_{k}^{i}$ to replace the k-th training sample $x_{k}^{i}$ of categorical feature i.

Assuming the training dataset is denoted as $D=\{{\left(x_{k},y_{k}\right)}_{k=1\ldots n}\}$, where $x_{k}=\left(x_{k}^{1},\ldots ,x_{k}^{m}\right)$ is a vector of m features, and $y_{k}\in \mathbb{R}$ is the target. CatBoost introduces a random permutation σ of training samples; for each sample, it uses Equation (5) to compute its TS, called Ordered TS [7]:

(5) $${\hat{x}}_{k}^{i}=\frac{\sum_{x_{j}\in D_{k}}I_{\left\{x_{j}^{i}=x_{k}^{i}\right\}}y_{j}+ap}{\sum_{x_{j}\in D_{k}}I_{\left\{x_{j}^{i}=x_{k}^{i}\right\}}+a}$$

where a>0 is a parameter; p is the average target value in the dataset. For the training sample, $D_{k}=\{x_{j}:\sigma \left(j\right)<\sigma \left(k\right)\}$, and $D_{k}=D$ for the test sample.

#### 4.3.2. Ordered Boosting

Assume the goal of learning is to train a minimal expected loss ℒ(F):=EL(y,F(x)), where L(·,·) is a smooth loss function, and (x,y) is the sample of the test dataset. The gradient boosting algorithm takes greedy fashion to construct an approximate sequence $F^{t}:{\mathbb{R}}^{m}\to \mathbb{R}$ by modifying from the previous estimate, as shown in Equation (6):

(6) $$F^{t}=F^{t-1}+\alpha h^{t}$$

where α is the step size; t=1,2,…; function $h^{t}:{\mathbb{R}}^{m}\to \mathbb{R}$ (the base learner) is chosen from a family of functions H to minimize the expected loss, as shown in Equation (7):

(7) $$h^{t}=argmin_{h\in H}ℒ\left(F^{t-1}+h\right)=argmin_{h\in H}EL\left(y,F^{t-1}\left(x\right)+h\left(x\right)\right)$$

Usually, the least-squares approximation is used, as shown in Equation (8):

(8) $$h^{t}=argmin_{h\in H}E{\left(-g^{t}\left(x,y\right)-h\left(x\right)\right)}^{2}$$

where $g^{t}\left(x,y\right):=\frac{\partial L\left(y,s\right)}{\partial s}{|}_{s=F^{t-1}\left(x\right)}$. However, in practice, the expectation in Equation (8) is unknown and is usually approximated using the same dataset D, as shown in Equation (9):

(9) $$h^{t}=argmin_{h\in H}\frac{1}{n}\overset{n}{\underset{k=1}{\sum }}{\left(-g^{t}\left(x_{k},y_{k}\right)-h\left(x_{k}\right)\right)}^{2}$$

This inevitably leads to a deviation between the base learner $h^{t}$ defined by Equation (9) and the solution of Equation (8), because the conditional distribution of the gradient $g^{t}\left(x_{k},y_{k}\right)|x_{k}$ is shifted from $g^{t}\left(x,y\right)|x$. The solution in CatBoost is called Ordered Boosting. It takes one random permutation σ of the training examples and maintains n different models $M_{1},\ldots ,M_{n}$, where model $M_{i}$ is learned using only the first i samples in the permutation. In each step, the model $M_{j-1}$ is used to calculate the residual of the j sample.

### 4.4. SHAP

Traffic safety is more concerned about how to interpret the model. However, most of the previous studies focused on improving the accuracy of the model and model comparison but neglected the interpretability. This paper applies SHAP model to interpret the model output. SHAP is an additive interpretation model inspired by Shapely value from game theory. It calculates the Shapely value of each feature, which is used as a basis for measuring the impact of the feature on the final output, as shown in Equation (10).

(10) $$g\left(z\right)=\phi_{0}+\overset{M}{\underset{j=1}{\sum }}\phi_{j}{z_{j}}^{\prime }$$

where:

g is the explanation model.

M is the number of features in the model.

$\phi_{j}$ is the SHAP value for the feature j.

${z_{j}}^{\prime }=1$ if the feature j is present, and otherwise, ${z_{j}}^{\prime }=0$.

$\phi_{0}$ is a constant.

The SHAP value for feature j is calculated by comparing the model output with and without the feature, described in the Equation (11):

(11) $$\phi_{j}=\underset{S\subseteq M\setminus \left\{j\right\}}{\sum }\frac{\left|S\right|!\left(\left|M\right|-\left|S\right|-1\right)!}{\left|M\right|!}\left[v\left(S\cup \left\{j\right\}\right)-v\left(S\right)\right]$$

where S is the subset of features used in the model; M is the set of all features; v(S∪{j}), and v(S) are the model output with and without feature j. If the SHAP value of a feature is positive, it indicates that the feature has a positive effect on the model results, and in this study, it tends to aggravate the severity of accidents. If the SHAP value is negative, it is the opposite.

However, the limitation of this model is that as the number of features increases, the computation cost increases exponentially. To break through this limitation, Lundberg et al. [46] proposed the TreeExplainer, which is suitable for tree-based machine learning models, such as LightGBM and CatBoost. The TreeExplainer can calculate the accurate Shapley value and correctly estimate the Shapley value when there is correlation between features [47]. The SHAP interaction values can be calculated as the difference between the Shapley values of feature i with and without feature j, as shown in Equation (12).

(12) $$\phi_{i,j}=\underset{S\subseteq M\setminus \left\{i,j\right\}}{\sum }\frac{\left|S\right|!\left(\left|M\right|-\left|S\right|-2\right)!}{\left|M\right|!}\left[v\left(S\cup \left\{i,j\right\}\right)-v\left(S\cup \left\{i\right\}\right)-v\left(S\cup \left\{j\right\}\right)+v\left(S\right)\right]$$

### 4.5. Performance Measures

**Figure 3:**
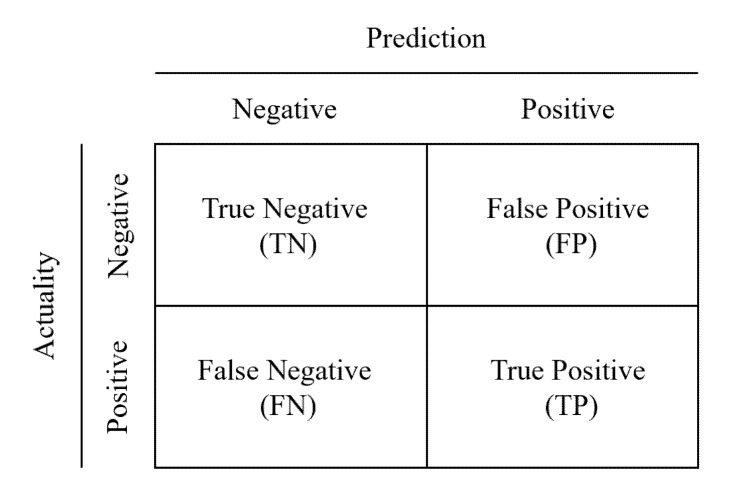
Confusion matrix.

The performance of machine learning models can be evaluated by several metrics, which can be generally calculated from the confusion matrix, depicted in Figure 3.

A common measure of model performance is the accuracy, where the total number of correct predictions is divided by the total number predictions. However, in unbalanced data sets, this metric cannot truly reflect the performance of the model. For example, assuming that the ratio of samples numbers of class A and class B in the test set is 9:1, the accuracy of the model that directly predicts all test samples are class A will be as high as 90%, but the performance of this model is very poor. To address this issue, the $F_{1}$ score is often used. $F_{1}$ score combines precision and recall and is computed as the harmonic mean of precision and recall, as shown in Equation (13).

(13) $$F_{1}=2*\frac{Precision*Recall}{Precision+Recall}$$

where $Precision=\frac{TP}{TP+FP}$ and $Recall=\frac{TP}{TP+FN}$. It is generally believed that the larger the $F_{1}$ score, the higher the performance of the model.

Another method is to use ROC (Receiver Operating Characteristic) as a measurement metric. The ROC curve is plotted with TPR (True-Positive Rate) against the FPR (False-Positive Rate), where TPR is on the *y*-axis and FPR is on the *x*-axis. The performance of the model can be intuitively judged by calculating AUC (Area Under ROC Curve). Generally, the value of AUC is between 0.5 and 1, with larger AUC representing better performance.

## 5. Results and Discussions

### 5.1. Model Parameters

Hyper-parameters tuning is the key step of training/fitting machine learning model. Proper parameters can improve the generalization performance, avoid overfitting, and reduce the complexity of the model. For the CatBoost model, several hyper-parameters listed in Table 1 need to be tuned. GridSearch method is the common method for hyper-parameters tuning in machine learning, but the disadvantage of this method is that it takes long time. In this paper, an open-source library named Hyperopt [48] is used for hyper-parameters tuning. It is an implementation based on Bayesian hyper-parameters optimization that optimizes continuous, discrete, and condition variables and automatically obtains the best hyper-parameters. Hyperopt is widely used in hyper-parameter tuning of machine learning model, which has a good performance [49,50,51].

In this study, 65% of the randomly selected data was used to train the model, and 35% of the data was used to test the model. In addition, a 10-fold cross validation is conducted on the training set to identify the optimal hyper-parameters for the CatBoost model. Three CatBoost models are developed for Group 1, Group 2, and Group 3, and the optimal hyper-parameter values are provided in Table 1. All experiments were processed in DataSpell (2021.3 EAP 20) using python 3.8.10, AMD Ryzen 7 4800U with Radeon Graphics, 1.80 GHz. Regarding the libraries, we used xgboost 1.5.0, lightgbm 3.3.1, catboost 1.0.3, scikit-learn 1.0.1, imbalance-learn 0.8.1, hyperopt 0.2.6, and shap 0.40.0.

**Figure 4:**
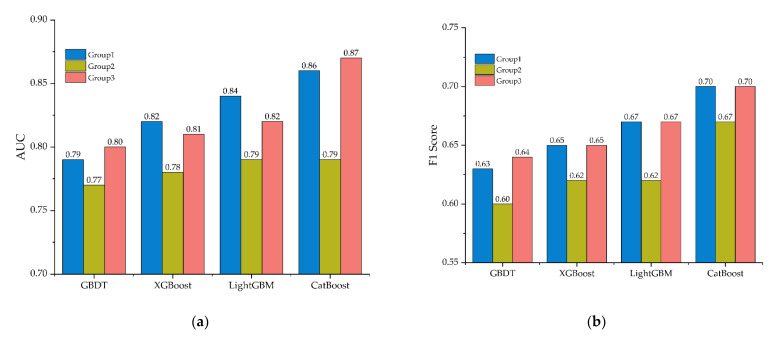
Classification performance: (**a**) AUC; (**b**) $F_{1}$ score.

As shown in Figure 4, the AUC values of CatBoost in the three groups are 0.86, 0.79, and 0.87, which indicates that CatBoost has better performance than GBDT (0.79, 0.77, and 0.80), XGBoost (0.82, 0.78, and 0.81), and LightGBM (0.84, 0.79, and 0.82). Similarly, the $F_{1}$ score values of CatBoost (0.70, 0.67, and 0.70) are better than other models. These encouraging AUCs and $F_{1}$ scores give a statistical proof of the excellent classification performance of the CatBoost in this study.

### 5.2. Feature Analysis

In this section, the interpreter of CatBoost output results is constructed by using the SHAP model, and the two questions mentioned above will be discussed in detail: what are the major factors causing serious accident outcomes for drivers of different driving experience? Are there any significant variations in their impacts?

**Figure 5:**
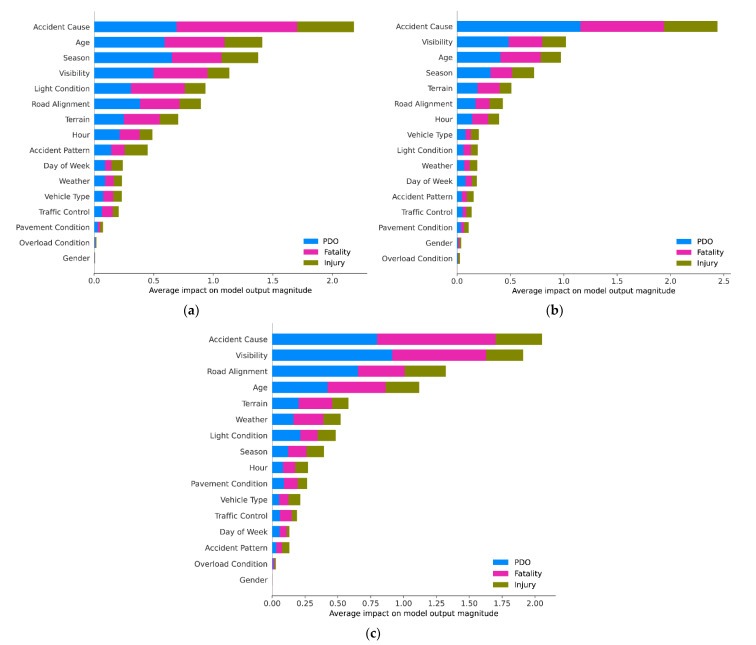
Feature importance on accident severity: (**a**) Group 1; (**b**) Group 2; (**c**) Group 3.

Figure 5 illustrates the average absolute impact of each feature on the model output magnitude, and the different colors indicate the different severity levels of accident. As shown in Figure 5a, accident cause is the strongest predictor for accident severity of novice drivers. Besides, age, season, visibility, light condition, road alignment, and terrain also have significant impact on accident outcomes. On the other hand, pavement surface condition, overload condition, and gender have the least impact on accident severity.

Regarding factors affecting accident severity of drivers in Group 2 (Figure 5b), accident cause is the strongest predictor, followed by visibility, age, season, terrain, and road alignment. Meanwhile, pavement surface condition, gender, and overload condition have the least impact on accident severity.

For drivers with more than 10 years of driving experience, as shown in Figure 5c, accident cause is also the strongest predictor. Visibility, road alignment, age, terrain, and weather have significant impact on accident severity. In addition, accident pattern, overload condition and gender have the least impact on accident severity.

**Figure 6:**
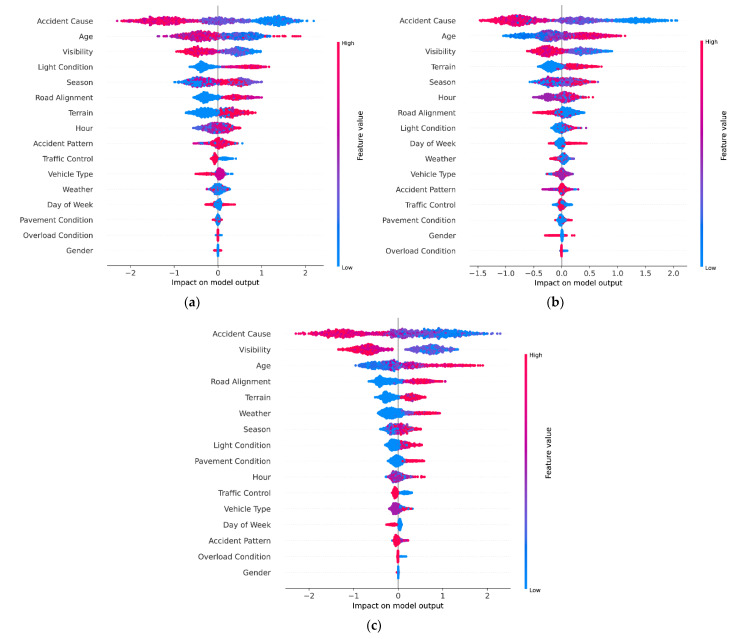
SHAP summary plots of fatal accident: (**a**) Group 1; (**b**) Group 2; (**c**) Group 3.

In accident prevention, it is necessary to understand how features affect fatal accident. This requires more information beyond feature importance. Figure 5 can only show which features are important; therefore, the SHAP summary plot is required for analysis. The summary plots of the CatBoost prediction result made by SHAP are shown in Figure 6. Each field represents the impact of the features on the probability of fatal accident of each group. The SHAP values sort the features’ rank on the left side of the *y*-axis and the *x*-axis is the scale of all samples calculated SHAP values. The color bar provides more details regarding how each feature affects the fatal accident. Each dot in the figure represents a data sample and is colored by the value of the feature from low (blue) to high (red).

As shown in Figure 6, accident cause, age, visibility, light condition, and terrain have a significant impact on fatal accident for both novice and experienced drivers. Accident cause is the most important feature affecting the occurrence of fatal accident. For drivers with different driving experience, the impact of accident cause is similar. In Figure 6, accident cause with high number (e.g., improper operation, illegal overtaking, illegal U-turn) decreases the risk of fatal accident, while accident cause with low number (e.g., overload or oversize, speeding, drowsy driving) correspondingly increases the risk. Besides, low visibility and poor light condition have positive SHAP values, which means that these features increase the risk of fatal accident. This is because the higher the visibility or the better the road light condition, the greater the sight distance of the driver, and the higher the safety level, as the driver can get sufficient time and distance to deal with emergencies. The result is consistent with Ahangar’s research [52]. For terrain, a previous study found a strong association between unfavorable terrain and locations with high accident rates, which in general continue to increase as horizontal curvature increases [53]. We also get similar results: mountains or hills can increase the risk of fatal accidents.

Different from the above features, the impact of age on fatal accidents is different for novice and experienced drivers. The risk of fatal accident is increased for young novice drivers and decreased for old novice drivers. This is consistent with Xiao’s study [4]. They found that increasing age reduces the risk of fatal accidents for novice drivers. However, for experienced drivers, the impact of age on fatal accident is opposite. At present, there are few studies on age in the field of accident severity of experienced drivers, and our findings supplement relevant studies to some extent. It also confirms the necessity of analyzing the influencing factors of accident severity for drivers with different driving experience.

### 5.3. Feature Dependency Analysis

**Figure 7:**
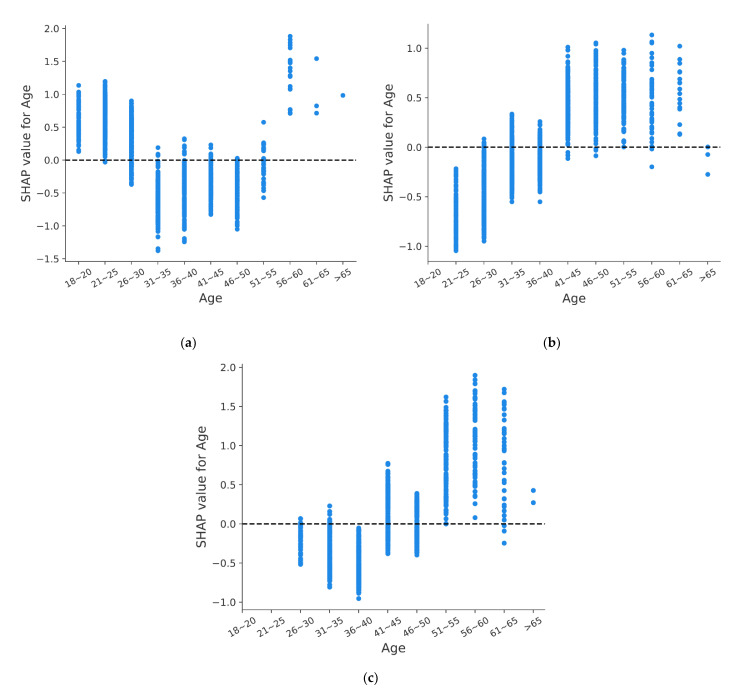
SHAP dependency plots of Age: (**a**) Group 1; (**b**) Group 2; (**c**) Group 3.

To analyze this variation further, the impact of age on fatal accident is analyzed separately. In Figure 7, the horizontal axis represents the value of feature, and the left vertical axis is for SHAP value, which describes the contribution of the corresponding feature to the CatBoost model output.

As shown in Figure 7a, for novice drivers, the trend of the impact of age on fatal accident presents a U shape. This shows that novice drivers younger than 30 or older than 55 are prone to suffer fatal accidents, which is consistent with previous research results [54,55]. One possible explanation is that novice drivers are more likely to suffer fatal accident due to the lack of driving experience [56]. Besides, young drivers are more likely to take risks [57], and older drivers’ driving ability decreases due to aging [58], which increases the risk of fatal accident in these two groups.

For experienced drivers, the impact of age on fatal accident is different from that of novice drivers. The SHAP value shows an obvious increasing trend with the increase of age. In Figure 7b,c, SHAP value increases from negative to positive. This indicates that for experienced drivers, the risk of fatal accident decreases when they are young and increases when they are old. For Group 2, SHAP value is positive when the age is over 40, while for Group 3, SHAP value is significantly greater than 0 when the age is over 50. This shows that the increase of driving experience reduces the impact of the increase of age on the risk of fatal accident.

From the above analysis, it can be found that, unlike young novice drivers, the risk of fatal accident of young experienced drivers is decreased because the increase of driving experience enables young drivers to deal with most emergencies on the road and reduce the risk of fatal accident. Although older experienced drivers have some driving experience, the decline of visual function and cognitive ability makes old drivers’ driving ability decline, which makes older drivers prone to suffer fatal accidents [59,60,61].

### 5.4. Feature Interaction Analysis

**Figure 8:**
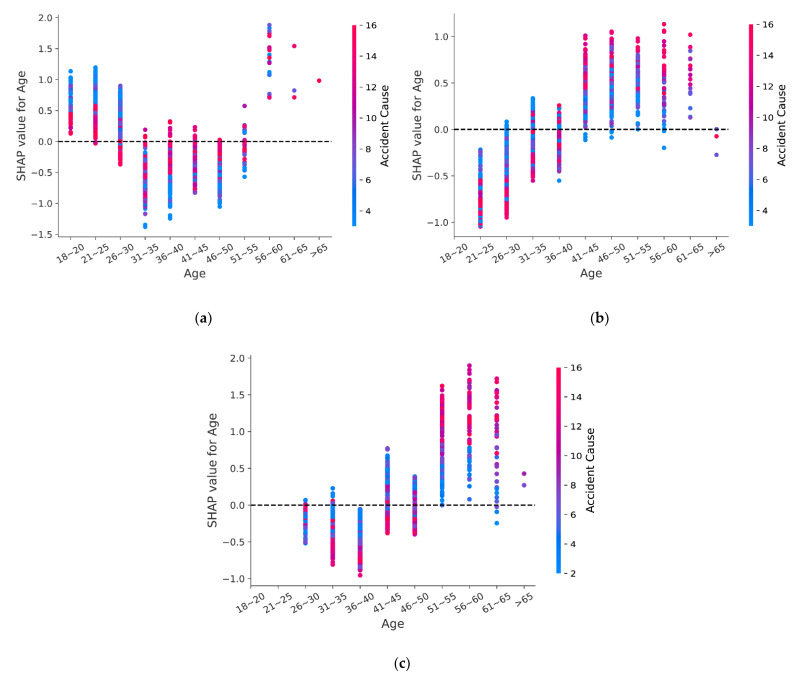
SHAP interaction effects plots: (**a**) Group 1; (**b**) Group 2; (**c**) Group 3.

Considering that accident cause is the strongest predictor of accident severity, meanwhile, it also contains the information of drivers’ condition. This paper provides an explanation for this variation by analyzing the interaction between accident cause and age. In Figure 8, the horizontal axis is the value of age, the left vertical axis is the SHAP value of age, and the right vertical axis is the value of accident cause.

As shown in Figure 8a, novice drivers younger than 30 years old are more likely to cause serious accidents due to accident cause with low number, such as overload, speeding, and drowsy driving. For experienced drivers with less than 10 years of driving experience, Figure 8b shows that drivers younger than 40 years old are more likely to suffer serious injuries due to an accident cause with a low number, while for drivers older than 40, the accident cause with a high number is more likely to lead to fatality. The overall trend of Figure 7c is the same as that of Figure 8b; the difference is that for drivers aged 40~50 with sufficient driving experience, an accident cause with a low number tends to bring serious outcomes, and an accident cause with a high number is less likely to lead to a fatal accident.

One possible explanation is that young drivers are more likely to get involved in fatal accidents due to risky behavior [1,62,63], and part of the reason for young drivers’ risk-taking behavior is that they cannot identify the potential hazards in the environment and choose inappropriate behavior [57]. Eye scanning pattern analysis showed that young drivers tend not to scan potential risk areas [64], and one study showed that once young drivers identify a hazard, it is difficult for them to deal with [65]. In addition, compared with experienced drivers, novice drivers are prone to engage in dangerous driving behavior and are more aggressive when driving [66], making young novice drivers more prone to suffer fatal accidents. Driving experience can not only help drivers accurately judge the driving environment but also reduce the risk of dangerous situations by reducing the tension of drivers when emergencies occur [67]. With the increase of driving experience, the risk of fatal accidents caused by dangerous behaviors of young drivers decreases.

For older drivers, the risk of fatal accident is increased due to improper operation, illegal overtaking, and other causes, and the increase of driving experience does not significantly reduce the risk. The possible reason is that with the increase of age, the physical function gradually decreases, and the vision, hearing, and response ability to the driving environment are weakened [67], which puts older drivers at risk of more serious injuries [59,60]. Additionally, compared with young drivers, older drivers tend to have a prudent driving style and less risk-taking behavior with the increase of age. It is worth noting that older drivers are inherently prone to be seriously injured in an accident because they are frail [68]. The higher risk of fatality among old drivers tends to reflect their physical vulnerability rather than the severity of the accident [60].

## 6. Conclusions

This research studied the influencing factors of traffic accident severity for drivers with different driving experience. Our innovation further analyzed the combined influence of age and driving experience. Three CatBoost models were developed and compared based on driving experience, and the output results were interpreted by using SHAP model. The following conclusions can be drawn:

1. In the analysis of influencing factors of accident severity, CatBoost generates the best result (AUC: 0.86, 0.79, and 0.87; *F*_1_ score: 0.70, 0.67, and 0.70), indicating the application potential of the model in traffic safety.
2. Accident cause, age, visibility, light condition, season, road alignment, and terrain are the key factors affecting the severity of traffic accident. Pavement surface condition, overload condition, accident pattern, and gender have the least impact on accident severity. The importance of these features varies for drivers with different driving experience in terms of accident severity.
3. The impact of age on fatal accidents is different for drivers with different driving experience. Novice drivers younger than 30 or older than 55 are prone to suffer fatal accidents, but for experienced drivers, the risk of fatal accident decreases when they are young and increases when they are old.

In the subsequent research, some limitations in this study can be solved. Firstly, satellite image data can be used to obtain accident-related features to expand the database, such as curve, number of lanes, etc., and these features would help to reflect the real accident information more specifically. Secondly, according to the inherent attributes of driving experience and age, it is possible to combine them into a new feature to further study their combined influence. In addition, considering that the accident characteristics have obvious regional attributes, the accident data of different regions can be utilized in the follow-up study.

## Figures and Tables

**Table 1:** CatBoost parameter tuning results.

Parameter	Description	Group 1	Group 2	Group 3
l2_leaf_reg	Coefficient at the L2 regularization term of the cost function.	2	5	5
learning_rate	Used for reducing the gradient step.	0.15	0.3	0.25
depth	Depth of the tree.	8	10	10
iterations	The maximum number of trees that can be built.	1000	400	500
loss_function	The metric to use in training.	MultiClass	MultiClass	MultiClass
od_wait	The number of iterations to continue the training after the iteration with the optimal metric value.	12	16	14

## Data Availability

Readers can assess the data from this email: chenshuaiming@chd.edu.cn.

## Appendix A

**Table A1:** Independent variables of traffic accident severity.

Variable	Description	Group1		Group2		Group3	
		*N*	%	*N*	%	*N*	%
Day of Week	Weekday = 1	1928	73.93%	2626	72.90%	1627	72.73%
	Weekend = 2	680	26.07%	976	27.10%	610	27.27%
Season	Spring: Match to May = 1	680	26.07%	922	25.60%	578	25.84%
	Summer: June to August = 2	659	25.27%	904	25.10%	553	24.72%
	Autumn: September to November = 3	649	24.88%	867	24.07%	526	23.51%
	Winter: December to February = 4	620	23.77%	909	25.24%	580	25.93%
Hour	0:00~06:59 = 1	216	8.28%	314	8.72%	199	8.90%
	07:00~09:59 = 2	414	15.87%	550	15.27%	368	16.45%
	10:00~15:59 = 3	923	35.39%	1280	35.54%	774	34.60%
	16:00~19:59 = 4	738	28.30%	982	27.26%	589	26.33%
	20:00~23:59 = 5	317	12.15%	476	13.21%	307	13.72%
Accident Cause	Overloaded or oversized = 1	54	2.07%	63	1.75%	57	2.55%
	Driving a vehicle that does not satisfy normal driving requirements = 2	70	2.68%	68	1.89%	84	3.76%
	Speeding = 3	620	23.77%	791	21.96%	368	16.45%
	Drowsy driving = 4	30	1.15%	33	0.92%	77	3.44%
	Traffic signal violation = 5	31	1.19%	51	1.42%	59	2.64%
	Driving without license = 6	46	1.76%	94	2.61%	55	2.46%
	Failing to give way to pedestrians or vehicles as required = 7	488	18.71%	670	18.60%	404	18.06%
	Reversing illegally = 8	38	1.46%	75	2.08%	51	2.28%
	Improper backing = 9	158	6.06%	224	6.22%	135	6.03%
	Illegal parking = 10	38	1.46%	49	1.36%	71	3.17%
	Affecting normal driving when changing lanes = 11	117	4.49%	186	5.16%	126	5.63%
	Improper operation = 12	178	6.83%	237	6.58%	95	4.25%
	Illegal overtaking = 13	121	4.64%	149	4.14%	170	7.60%
	Driving in a place not for traffic = 14	257	9.85%	410	11.38%	192	8.58%
	Illegal vehicle meeting = 15	191	7.32%	288	8.00%	148	6.62%
	Illegally cut in = 16	97	3.72%	118	3.28%	57	2.55%
	Illegal U-turn = 17	74	2.84%	96	2.67%	88	3.93%
Accident Pattern	The occupants dropped or thrown = 1	3	0.12%	6	0.17%	3	0.13%
	Crushing pedestrians = 2	53	2.03%	68	1.89%	50	2.24%
	Vehicle falling = 3	23	0.88%	29	0.81%	19	0.85%
	Vehicle rolled or rolled over = 4	71	2.72%	96	2.67%	56	2.50%
	Vehicle crashes into a non-fixed object = 5	3	0.12%	2	0.06%	2	0.09%
	Vehicle crashes into a fixed object = 6	48	1.84%	94	2.61%	55	2.46%
	Crashing into a stationary vehicle = 7	50	1.92%	100	2.78%	84	3.76%
	Other vehicle-to-vehicle accidents = 8	21	0.81%	24	0.67%	25	1.12%
	Scratch pedestrians = 9	317	12.15%	500	13.88%	281	12.56%
	Other vehicle-pedestrian accidents = 10	8	0.31%	7	0.19%	5	0.22%
	Crashing into a moving vehicle = 11	2011	77.11%	2676	74.29%	1657	74.07%
Weather	Sunny = 1	1882	72.16%	2618	72.68%	1607	71.84%
	Cloudy = 2	346	13.27%	476	13.21%	324	14.48%
	Foggy = 3	6	0.23%	8	0.22%	8	0.36%
	Rainy = 4	347	13.31%	469	13.02%	279	12.47%
	Snowy = 5	27	1.04%	31	0.86%	19	0.85%
Pavement Surface Condition	Dry = 1	2172	83.28%	2994	83.12%	1874	83.77%
	Wet = 2	379	14.53%	519	14.41%	309	13.81%
	Water standing = 3	38	1.46%	53	1.47%	33	1.48%
	Flooding = 4	2	0.08%	3	0.08%	3	0.13%
	Muddy = 5	2	0.08%	9	0.25%	1	0.04%
	Icy or snowy = 6	15	0.58%	24	0.67%	17	0.76%
Visibility	< 50 m = 1	411	15.76%	516	14.33%	349	15.60%
	50~99 m = 2	768	29.45%	1063	29.51%	661	29.55%
	100~200 m = 3	513	19.67%	698	19.38%	429	19.18%
	> 200 m = 4	916	35.12%	1325	36.79%	798	35.67%
Traffic Control	Without signal control = 1	729	27.95%	1049	29.12%	602	26.91%
	With signal control = 2	1879	72.05%	2553	70.88%	1635	73.09%
Light Condition	Day = 1	1731	66.37%	2365	65.66%	1453	64.95%
	Dawn = 2	21	0.81%	41	1.14%	24	1.07%
	Dusk = 3	40	1.53%	80	2.22%	53	2.37%
	Dark: streetlight on = 4	355	13.61%	493	13.69%	301	13.46%
	Dark: streetlight off = 5	461	17.68%	623	17.30%	406	18.15%
Terrain	Plain = 1	1561	59.85%	2127	59.05%	1338	59.81%
	Hill = 2	208	7.98%	265	7.36%	170	7.60%
	Mountain = 3	839	32.17%	1210	33.59%	729	32.59%
Road Alignment	Straight and level = 1	1657	63.54%	2322	64.46%	1447	64.68%
	Straight with gradient = 2	68	2.61%	103	2.86%	65	2.91%
	Curved and level = 3	339	13.00%	438	12.16%	258	11.53%
	Curved with gradient = 4	544	20.86%	739	20.52%	467	20.88%
Gender	Male = 1	2476	94.94%	3390	94.11%	2127	95.08%
	Female = 2	132	5.06%	212	5.89%	110	4.92%
Age	18~20 = 1	110	4.22%	0	0.00%	0	0.00%
	21~25 = 2	541	20.74%	266	7.38%	0	0.00%
	26~30 = 3	485	18.60%	746	20.71%	49	2.19%
	31~35 = 4	383	14.69%	711	19.74%	317	14.17%
	36~40 = 5	411	15.76%	620	17.21%	514	22.98%
	41~45 = 6	334	12.81%	569	15.80%	511	22.84%
	46~50 = 7	202	7.75%	345	9.58%	419	18.73%
	51~55 = 8	99	3.80%	221	6.14%	247	11.04%
	56~60 = 9	36	1.38%	90	2.50%	119	5.32%
	61~65 = 10	6	0.23%	31	0.86%	58	2.59%
	>65 = 11	1	0.04%	3	0.08%	3	0.13%
Overload Condition	Overloaded = 1	205	7.86%	232	6.44%	149	6.66%
	Not overloaded = 2	2403	92.14%	3370	93.56%	2088	93.34%
Vehicle Type	Trailer = 1	196	7.52%	208	5.77%	122	5.45%
	Tractor = 2	43	1.65%	49	1.36%	39	1.74%
	Automobile = 3	1955	74.96%	2723	75.60%	1698	75.91%
	Motorcycle = 4	394	15.11%	603	16.74%	363	16.23%
	Other = 5	20	0.77%	19	0.53%	15	0.67%
